# Intraarticular vs. extraarticular ropivacaine infusion following high-dose local infiltration analgesia after total knee arthroplasty: A randomized double-blind study

**DOI:** 10.3109/17453674.2011.625535

**Published:** 2011-11-25

**Authors:** Igor Dobrydnjov, Christian Anderberg, Christer Olsson, Olga Shapurova, Krister Angel, Stefan Bergman

**Affiliations:** ^1^Department of Rheumasurgery; ^2^Research and Development Centre, Spenshult Hospital, Oskarström, Sweden

## Abstract

**Background and purpose:**

Ropivacaine infusion following high-volume local infiltration analgesia has been shown to be effective after total knee arthroplasty, but the optimum site of administration of ropivacaine has not been evaluated. We compared the effects of intraarticular and extraarticular adminstration of the local anesthetic for postoperative supplementation of high-volume local infiltration analgesia.

**Patients and methods:**

In this double-blind study, 36 rheumatic patients aged 51–78 years with physical status ASA 2–3 who were scheduled for total knee arthroplasty were randomized into 2 groups. All patients received wound infiltration at the end of surgery with 300 mg ropivacaine, 30 mg ketorolac, and 0.5 mg epinephrine (total volume 156 mL). A tunneled catheter was randomly placed either extraarticularly or intraarticularly. Continuous infusion of ropivacain (0.5%, 2 mL/h) was started immediately and was maintained during the next 48 h. Pain intensity at rest, on movement, and with mobilization was estimated by the patients and the physiotherapist; rescue morphine consumption was recorded.

**Results:**

As estimated by the patients, ropivacaine administered intraarticularly did not improve analgesia relative to extraarticular infusion, but improved the first mobilization. The incidence of high intensity of pain (VAS 7–10) was less in the group with intraarticular infusion. Analgesic requirements were similar in the 2 groups (47 mg and 49 mg morphine). No complications of postoperative wound healing were seen and there were no toxic side effects.

**Interpretation:**

Continuous infusion of ropivacaine intraarticulary did not improve postoperative analgesia at rest relative to extraarticular administration, but it appeared to reduce the incidence of high pain intensity during first exercises, and could therefore be expected to improve mobilization up to 24 h after total knee arthroplasty.

Pain after total knee arthroplasty is severe in two-thirds of patients (Bonica 1984, Beattie et al. 1997). The pain may be a result of trauma to the bone or soft tissues or a result of hyperperfusion after tourniquet release (Estebe et al. 1995). The optimal form of pain relief is one that is applied preoperatively, perioperatively, and postoperatively to avoid the establishment of pain hypersensitivity (Badner et al. 1996). Good pain relief allows effective postoperative rehabilitation (Shoji et al. 1990). In contrast to epidural analgesia and femoral block, an alternative method to achieve good postoperative pain relief is local infiltration combined with single-shot injection or continuous infusion of local anesthetics into the surgical site. Local anesthetic infiltration is helpful in the management of postoperative pain after several orthopedic procedures (De Andres et al. 1993). The use of intraarticular analgesia to limit postoperative pain following knee arthroplasty has been investigated, with different results (Smith et al. 1991, Allen et al. 1993, Osborne and Keene 1993, Badner et al. 1996, Mauerhan et al. 1997, Ritter et al. 1999).

A local infiltration analgesia (LIA) technique was developed by Kerr and Kohan in Sydney, Australia (Rostlund and Kehlet 2007, Kerr and Kohan 2008). With this technique, the long-acting local anesthetic ropivacaine, a non-steroidal anti-inflammatory drug (ketorolac), and epinephrine are infiltrated periarticulary during surgery. An alternative technique that might have widespread applicability is the insertion of catheters to allow continuous infusion of local anesthetics into the surgical wound at the end of the procedure. There is a need for detailed systematic studies to evaluate the optimal site of administration of local anaesthetics. In this randomized, double-blind study, we compared the analgesic effects of continuous infusion of local anesthetics either intraarticulary or extraarticulary after TKA using the LIA technique.

## Patients and methods

The study protocol was approved by the regional ethics committee (May 14, 2009, FOUS09002) and the Swedish Medical Products Agency, and was conducted in accordance with the Declaration of Helsinki (ClinicalTrials identifier: NCT01050738). Written informed consent was obtained from each patient before the start of the study.

50 patients with rheumatoid arthritis who were scheduled for total knee arthroplasty were screened for eligibility. Inclusion criteria were age < 80 years, a weight of 50–120 kg, and an ability to provide informed consent for the study and to cooperate with study personnel. Exclusion criteria were major psychological problems, allergies to any of the ingredients of the injection, renal insufficiency, abnormal liver enzymes, a history of stroke or a major neurological deficit, uncontrolled angina, chronic opioid use, severe diabetes, inability to understand or use patient controlled analgesia (PCA), or any contraindications for spinal anesthesia. Elective unilateral total knee arthroplasty was performed at the Unit for Rheumatic Surgery, Spenshult Hospital from September 2009 through March 2010 and patients were followed for 3 months after surgery.

### Randomization and blinding

**Figure 1.**
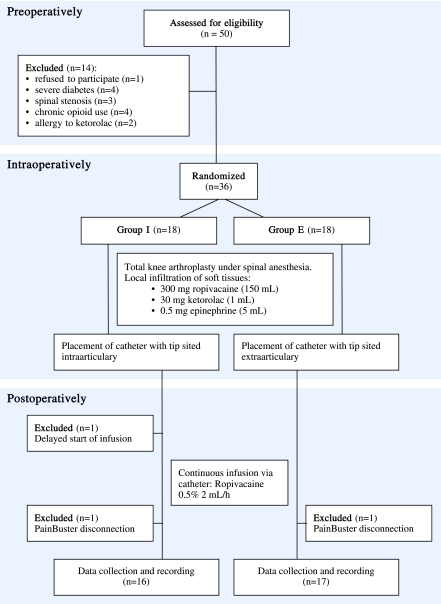
Flow chart of the study.

50 patients were assessed for eligibility and 14 were excluded before randomization (Figure 1). The 36 patients were randomly allocated to 1 of 2 treatment groups, each comprising 18 patients, by using computer-generated random numbers inserted into sealed envelopes marked 1–36. The randomization envelope was opened a few minutes before the start of surgery.

The patients, the 2 independent study investigators, the study physiotherapist, and all the staff concerned with the postoperative care of the patients were blinded. The operating surgeons who were not blinded did not take any part in patient care after completion of the operation. The key-list regarding randomization was opened only after the data from the last patient had been collected and recorded.

### Anesthesia

All the patients received paracetamol (1 g) orally before planned surgery. A midline lumbar puncture was performed at the L3–L4 interspace with a 27-G Quincke spinal needle, with the patients in a sitting position, using 20 mg of plain bupivacain (Marcain Spinal; Astra, Södertalje, Sweden). Noninvasive blood pressure was measured intraoperatively at 5-min intervals and heart rate (HR), respiratory rate, and SpO2 were registered continuously using Datex-Engstrom AS/3. Hypotension was defined as a decrease in systolic blood pressure by 30% from the baseline value, and was treated with ephedrine (5 mg intravenously). Bradycardia was defined as HR < 50 beats/min, and was treated with atropine (0.5 mg intravenously). Intraoperative sedation was provided with propofol intravenously, with a target plasma concentration of 0.5–0.8 µg/mL according to the patient's needs. Intravenous fluids were administered in accordance with estimated blood loss.

Kloxacillin (2 g) was given intravenously before surgery and twice postoperatively, with 8-h intervals. For thromboprophylaxis, rivaroxaban (10 mg), was administered orally once each evening for 14 days, starting on the day of surgery.

### Surgery

The knee arthroplasty was performed by 1 of 2 surgeons using a standard medial parapatellar approach. The same technique was used in both groups, and all patients received the NexGen CR prosthesis (Zimmer Sweden AB). A tourniquet was used in all patients.

### Pain management

At the end of the operation, the surgeon infiltrated a solution containing 300 mg ropivacaine (150 mL of 0.2% Narop (AstraZeneca, Sodertalje, Sweden), 30 mg Toradol (1 mL of Ketorolac; Roche AB, Stockholm, Sweden), and 0.5 mg epinephrine (5 mL of 0.01% adrenaline) into the soft tissues periarticularly in the following way. The first aliquot of 30 mL of the mixture was injected, just prior to implantation of the component, into the posterior aspect of the capsule and the medial and lateral collateral ligaments. 50 mL was injected into the deep tissues around the medial and collateral ligaments and the wound edges before suturing. The rest was injected into the capsule incision, the synovial sheet, the ligaments, and the subcutaneous tissue. Care was taken to avoid excessive infiltration in the area of the common peroneal nerve. Wound drains were not placed in any patients.

A multi-hole Perifix One catheter (B. Braun Medical AB, Danderyd, Sweden) was tunnelled laterally through muscle and skin, 6–8 cm from the edge of the wound. The tip of the catheter was placed according to randomization either in the distal part of the joint intraarticularly (the I group) or, in the case of an extraarticular location (the E group), the tip of the catheter was embedded into the soft tissues proximal to the patella. The medial split of the quadriceps tendon, the medial retinaculum, and the soft tissues medial to the patellar tendon were meticulously sutured with interrupted and continuous techniques, to prevent any communication between the joint space and the subcutaneous space. The catheter was connected to a pump (Pain Buster; I-Flow Corp., Lake Forest, CA). Thereafter, a continuous infusion of 5 mg/mL ropivacaine was started at 2 mL/h.

Postoperative pain was treated using paracetamol (1 g orally every 6 h) in all patients. Pain intensity was assessed by VAS (where 0 = no pain and 10 = worst pain). When the patient had a VAS score at rest of ≥ 3, morphine (1 mg) was repeatedly administered intravenously until the score was < 3. Then all patients were provided with a PCA device programmed to deliver morphine (a morphine bolus of 1 mg, a lock-out of 6 min, and a maximum of 10 mg/h) as a rescue medication for 48 h after surgery.

### Outcome measures

The consumption of patient-controlled analgesia was measured hourly during the 48-h postoperative period and the patient's overall analgesic consumption was measured to permit comparison between the 2 treatment groups. Patients used a visual analog scale to assess pain, both at rest and with motion.

All patients were mobilized by a physiotherapist 24 h after surgery and then at 12-h intervals. Pain during mobilization was judged by the patients according to VAS score and by the physiotherapist according to Verbal Rating Score (VRS: 0 = none, 1 = mild, 2 = moderate, 3 = strong, 4 = intense, and 5 = unbearable). Pain intensity and need of rescue opioids during mobilization were recorded.

During the first 12 h postoperatively, mean arterial pressure, heart rate, oxygen saturation, and sedation were recorded every 2 h and then every 6 h thereafter. Sedation was assessed on a 4-point VRS (1 = no sedation, 2 = light sedation, 3 = somnolence, and 4 = deep sedation). The physiotherapists assessed knee flexion postoperatively and recorded patients' ability to climb stairs (they were asked to climb a 4-step staircase without handrail support).

The appearance of symptoms of central nervous system toxicity, such as numbness of the tongue, dizziness, visual disturbances, metallic taste, tinnitus, muscular twitching and dysarthria, or hemodynamic changes, were criteria for immediate discontinuation of ropivacaine infusion.

Discharge was decided by the surgeons who were blinded regarding the analgesic treatment, according to the following discharge criteria: (1) satisfactory pain control for self-mobility; (2) uncomplicated wound-healing process; (3) uncomplicated clinical and radiographic outcome; (4) no evidence of deep vein thrombosis; and (5) acceptable level of hemoglobin and liver/kidney function; and (5) ability for active flexion ≥ 65 degrees.

### Statistics

Unless otherwise stated, the results are expressed as mean with 95% confidence interval or median (range). All normally distributed continuous variables were analyzed by one-way ANOVA followed by Tukey's test. The degree of sedation and analgesia in the groups was assessed using the Mann-Whitney U-test. If there were significant differences, the analysis was continued with post hoc comparisons of differences between pairs of groups using the Mann-Whitney U-test. Fisher's exact test was used to determine differences in all nominal data. Any p-value < 0.05 was considered statistically significant.

The aim was to determine whether either intraarticular or extraarticular administration of local anesthetics was superior to the other, with a null hypothesis of no difference between the two groups. Calculation of sample size was based on a minimum difference of 10 mm in the VAS measurement for pain between group means, based on a reported value of minimal clinically important differences in acute pain (DeLoach et al. 1998), on a standard deviation of 16, with p = 0.80 and α = 0.05. We obtained a sample size of 16 patients per group. A sample size of 18 patients per group was then chosen to ensure that the calculated number of patients would still be available by the final analysis.

## Results

Both groups were comparable with respect to age, height, body weight, ASA classification, time of operation and tourniquet release, dosage of local anesthetics and propofol, and time until the first dose of analgesic was applied. There were more females than males in both groups (Table). Subarachnoid block was successful for intraoperative analgesia in all cases. No additional analgesics were used during the operation.

**Table:** Demographic and intraoperative data on patients

	Group I (n=16)	Group E (n=17)	p-value
Age, years	70 (64–76) **^a^**	64 (55–73)	0.2
Sex (M/F)	6/10	3/14	0.2
ASA physical status (II/III)	15/1	17/0	0.5
Length, cm	177 (170–184)	174 (163–184)	0.7
Weigh, kg	74 (60–89)	83 (71–95)	0.3
Duration of operation, min	89 (75–104)	92 (65–119)	0.7
Intraoperative dose of propofol, mg	188 (156–220)	179 (139–219)	0.4
Time to the first morphine request, min	436 (274–618)	449 (235–663)	0.9

**^a^** Values with parentheses are mean (95% CI) Group I: intraarticular infusion of ropivacaine; group E: extraarticular infusion of ropivacaine; ASA physical status II: mild systemic disease; ASA physical status III: severe systemic disease.

3 patients were excluded from analysis. By mistake, 1 patient in the I group did not receive the continuous postoperative infusion because the start of the infusion was delayed. 1 patient in each study group was excluded because of catheter disconnection. Thus, 33 patients completed the study. Patient characteristics, duration of surgery, blood loss, and the intraoperative dose of propofol and ephedrine were similar in both groups with no statistically significant differences (Table).

### Primary endpoint, pain relief

**Figure 2.**
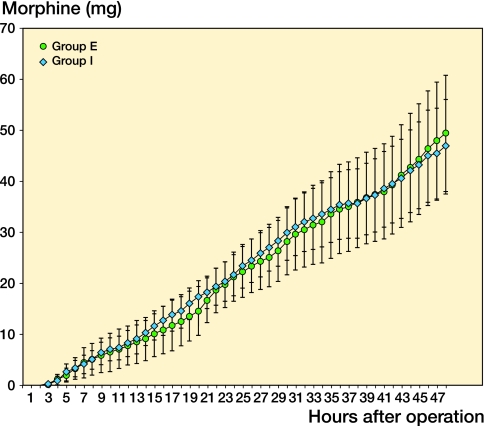
Cumulative postoperative morphine (mg; mean ± 95% CI) during the first 48 postoperative hours after TKA. There were no significant differences between group I (intraarticular infusion of ropivacaine) and group E (extraarticular infusion of ropivacaine).

The total amounts of PCA morphine required during the study period were compared for group I and group E. The overall analgesic consumption in rescue morphine was similar between the 2 groups: after 48 h, morphine consumption had reached 50 mg in both groups (Figure 2).

**Figure 3.**
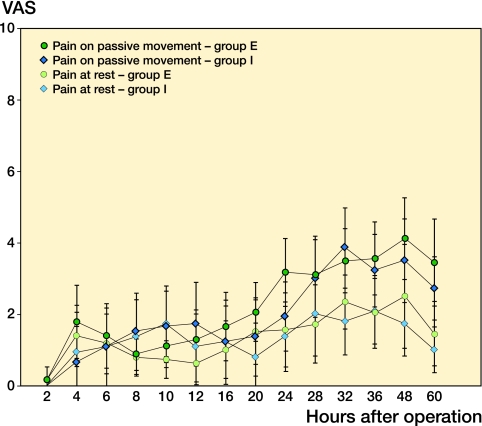
Mean postoperative visual analog pain score (VAS; mean ± 95% CI) assessed at rest and on passive movement at different times during the 48-h follow-up after TKA. No significant difference was recorded between groups.

There was no statistically significant difference in VAS score—at rest or with movement—between the groups (the lowest p-value being 0.07; Figure 3). The mean visual analog scale rating for pain intensity at rest was < 3 (out of 10) during all the time of the investigation and the highest pain score in movement was observed between 24 h and 60 h, when it reached a maximum rating of 6.

**Figure 4.**
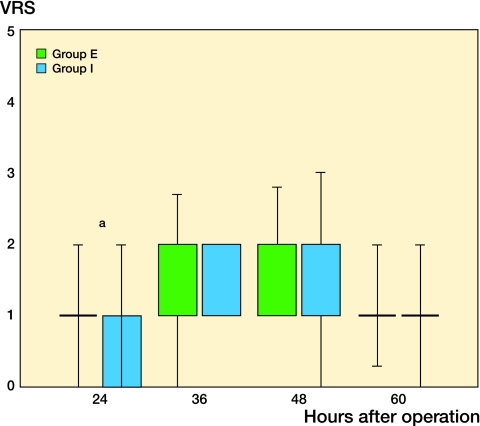
Box plots showing intensity of pain during exercise estimated by physiotherapists according to Verbal Rating Score (0 = none, 1 = mild, 2 = moderate, 3 = strong, 4 = intense, and 5 = unbearable) in group I (intraarticular infusion of ropivacaine) and group E (extraarticular infusion of ropivacaine). The boxes show the interquartile range, and the whiskers the minimum and maximum values.

There was greater physiotherapist satisfaction concerning the first mobilization in the I group (p < 0.05) (Figure 4). Patients in group I showed a trend toward lower mean scores during exercise at 48 h postoperatively (p < 0.07). 11 patients in the E group had severe pain at mobilization (VAS ≥ 7), as opposed to 3 patients in the I group (p = 0.03). The dose of morphine as rescue analgesic during mobilization was comparable between groups and there was no statistically significant difference during all the time of the study (p = 0.8).

### Secondary endpoints

Postoperative knee flexion and time to climb stairs were similar between the groups. No serious side effects (respiratory or cardiovascular suppression) were observed in any patient, but side effects of minor importance occurred in both groups. Dizziness was observed in more patients in the E group more often than in the I group (7 and 5 patients, respectively; p < 0.4). The frequency of other side effects such as nausea and pruritus was unaffected by the treatment modality throughout the study period.

No neurological or cardiac complications could be attributed to ropivacaine administration. No signs of local or systemic infections were noted in any of the patients, and wound healing was considered normal by the surgeon. The patients were ready for discharge after mean 70 h for the I group and 74 h for the E group. No patients were re-admitted for any complications during the 6 months of follow-up.

## Discussion

Although Andersen et al. (2008) reported that there was no difference in analgesic effect in administration of ropivacaine intraarticulary, or in the extraarticular wound space, our double-blind, randomized study has shown that postoperative mobilization after total knee arthroplasty can be improved by intraarticular application of local anesthetics. Our results may be due to a different design. We have not found any published studies evaluating the effect of continuous intraarticular infusion of local anesthetics after TKA.

Direct application of local anesthetic in wounds can provide analgesia through several mechanisms. Local anesthetics would directly block transmission of pain from nociceptive afferents from the wound surface. In addition, there is growing evidence that local anesthetic can inhibit local inflammatory responses to injury, which can sensitize nociceptive receptors and contribute to pain and hyperalgesia. For example, local anesthetics can reduce release of inflammatory mediators from neutrophils, reduce neutrophil adhesion to the endothelium, reduce formation of free oxygen radicals, and reduce edema formation (Hollmann and Durieux 2000, Hahnenkamp et al. 2002). Regardless of mechanisms, the ability to provide prolonged application of local anesthetics to wounds through a catheter is probably important, as previous systematic reviews have been unable to detect any appreciable benefit from single injection of local anesthetics in abdominal operation or laparoscopy (Moiniche et al. 1998, 2000). In the present study, ropivacaine infused intraarticulary did not reduce either mean VAS or morphine consumption, but the number of patients with a high level of pain intensity differed depending on the site of administration of local anesthetic.

Ropivacaine is pharmacologically similar to bupivacaine, but is associated with less cardiac and central nervous system toxicity, which allows patients to tolerate a larger dose (Mather et al. 2005, Mather 2010). Although the main action of ropivacaine is to block afferent peripheral nociceptive activity, the drug has also been shown to have some anti-inflammatory properties in human mucosal cells (Martinsson et al. 1997). Our patients received an initial dose of 300 mg ropivacaine followed by 10 mg/h, i.e. 540 mg of ropivacaine over the first 24 h (or altogether 780 mg in 48 h). One of several limitations in our study was that we did not check ropivacaine concentration in plasma. However, our dosages of ropivacaine were not higher than used in other studies. No toxic blood levels of ropivacaine were measured in the study by Busch et al. (2006) (using 400 mg ropivacaine for infiltration), or in the study by Vendittoli et al. (2006) (using 400 mg ropivacaine for infiltration and 150 mg for injection on the following day). None of the patients reported tinnitus, tingling, perioral numbness, or other toxic symptoms of local anesthetics. We used epinephrine intraoperatively, which reduces the toxicity of the local anesthetic by keeping it localized to the area of injection (Solanki et al. 1992). The maximum tolerated doses of local anesthetics with epinephrine administered intraarticulary or by infiltration have not been properly established (Rosenberg et al. 2004). The use of epinephrine in the ropivacaine injection may have reduced bleeding into the knee, but because neither group of patients had drains, there was no way of ascertaining whether any of the patients had reduced bleeding into the knee.

Our findings are consistent with the results of 2 recent studies in which patients were randomized to receive either periarticular or intraarticular treatment and patient-controlled analgesia (PCA) with morphine, or PCA alone (Busch et al. 2006, Vendittoli et al. 2006). Both studies assessed infiltration with ropivacaine, ketorolac, and epinephrine, though Busch et al. added epimorphine to the solution and Vendittoli et al. added a postoperative bolus through an intraarticular catheter. The study by Busch et al. included 64 patients who were blinded (as was the postoperative team), and the study by Vendittoli et al. included 42 patients. The positive results of these 2 studies are not surprising, as the analgesic treatment in the control groups mainly consisted of parenteral morphine.

According to previously published studies, the cumulative 24-h morphine consumption has been reduced from 40–60 mg (Sites et al. 2003, Stiller et al. 2007) to 30 mg (Vendittoli et al. 2006) by using LIA technique. In the present study, continuous postoperative infusion of ropivacaine in combination with LIA allowed a reduction of the 24-h dose of morphine, of up to 20 mg in both groups. Opioid-sparing provided objective evidence of efficacy of continuous wound catheters and probably contributed to reduced incidence of postoperative nausea and vomiting (PONV). With incidence ranging from 30% to 80% (Gan et al. 2003), PONV has been consistently rated by patients as a primary concern after operation that is more important than postoperative pain (Gan et al. 2003, Apfel et al. 2004). The patients in our study had a low incidence of PONV (4/33), which corresponds to a low dose of morphine. PONV can result in major patient discomfort, poor satisfaction, and increased economic burden, as it has been identified as a major cause of increased nursing care and delayed discharge.

Early mobilization is important after TKA. Effective exercises with physiotherapists reduce the risk of complications such as deep vein thrombosis, pulmonary embolism, pneumonia, and urinary retention (Beard et al. 2002). In our study, postoperative intraarticular infusion of ropivacaine was not clearly superior to extraarticular infusion during all mobilizations, but it gave improved pain relief 24 h after the operation.

The incidences of technical failure (2/33) or local anesthetic toxicity (0/33) from wound catheters were low in our study. One possible concern about this technique is the potential risk of delayed wound healing and infection. The size of our study does not allow conclusions to be drawn regarding the risk of deep knee infection. The data in the literature about this complication are controversial. Several authors have expressed potential concern about wound infections from the presence of a catheter. (Brown and Morrison 2004) reported that rates of superficial wound infection were similar between active (0.7%) and control groups (1.2%), while in another studies with the intraarticular catheter in place for 48 h, 55 h, and 72 h, no increased risk of infection was found (Bianconi et al. 2003, Rasmussen et al. 2004, Vendittoli et al. 2006).

One limitation of the present study was the low number of patients, and the results must be interpreted with this in mind. Positive findings at the 5% significance level should be interpreted with caution, due to multiple comparisons.

In conclusion, we found that continuous infusion of ropivacaine intraarticulary did not improve postoperative analgesia at rest compared to extraarticular infusion, but it appeared to reduce the incidence of high pain intensity during first exercises. It could therefore be expected to improve mobilization within 24 hours after total knee arthroplasty.
